# Latent Toxoplasmosis Effects on Rodents and Humans: How Much is Real and How Much is Media Hype?

**DOI:** 10.1128/mBio.02164-19

**Published:** 2020-03-17

**Authors:** Hannah J. Johnson, Anita A. Koshy

**Affiliations:** a Neuroscience Graduate Interdisciplinary Program, University of Arizona, Tucson, Arizona, USA; b Department of Immunobiology, University of Arizona, Tucson, Arizona, USA; c Bio5 Institute, University of Arizona, Tucson, Arizona, USA; d Department of Neurology, University of Arizona, Tucson, Arizona, USA; University of Texas Health Science Center at Houston

**Keywords:** *Toxoplasma gondii*, behavior, central nervous system infections, human, murine, neurodegeneration

## Abstract

Toxoplasma gondii is a ubiquitous, intracellular protozoan parasite with a broad range of intermediate hosts, including humans and rodents. In many hosts, T. gondii establishes a latent long-term infection by converting from its rapidly dividing or lytic form to its slowly replicating and encysting form. In humans and rodents, the major organ for encystment is the central nervous system (CNS), which has led many to investigate how this persistent CNS infection might influence rodent and human behavior and, more recently, neurodegenerative diseases. Given the interest in this topic, here we seek to take a global approach to the data for and against the effects of latent T. gondii on behavior and neurodegeneration and the proposed mechanisms that might underlie behavior modifications.

## Abstract (precis)

Toxoplasma gondii is a ubiquitous, intracellular protozoan parasite with a broad range of intermediate hosts, including humans and rodents. In many hosts, T. gondii establishes a latent long-term infection by converting from its rapidly dividing or lytic form to its slowly replicating and encysting form. In humans and rodents, the major organ for encystment is the central nervous system (CNS), which has led many to investigate how this persistent CNS infection might influence rodent and human behavior and, more recently, neurodegenerative diseases.

## INTRODUCTION

Toxoplasma gondii is a common, intracellular protozoan parasite in the Apicomplexa family and is found worldwide. While T. gondii only undergoes sexual reproduction in felids, most warm-blood animals, including humans and rodents, are natural intermediate hosts for T. gondii (1). Most intermediate hosts ingest T. gondii as either a sporozoite, which is contained in the oocysts shed by newly infected cats, or a bradyzoite, which is the tissue-persistent form of the parasite. Once ingested, parasites convert to the fast-replicating asexual form, the tachyzoite, which disseminates throughout the host and is controlled by the immune response. In certain organs (e.g., skeletal muscle or central nervous system [CNS]), T. gondii switches to its bradyzoite form, which is characterized by slow replication and encystment. Intracellular bradyzoite-filled cysts evade the immune response and have the potential to persist for the entire life of the host (2–5). Like other intermediate hosts, humans and rodents primarily become infected with T. gondii through ingestion of contaminated food and water, although humans can also pass T. gondii vertically or through organ transplantation (6). In humans and rodents, the major organ for encystment is the CNS (7, 8).

While T. gondii is established to cause symptomatic disease in immunocompromised hosts (e.g., toxoplasmic encephalitis in AIDS patients [9–12]) and occasionally in immunocompetent hosts (e.g., mono-like syndrome, chorioretinitis), the parasite’s ability to cause a latent long-term CNS infection has generated continued interest in understanding how this persistent CNS infection might influence host behavior and outcomes. The potential for parasite-induced behaviors is especially appealing given the recognition that other parasites cause host behavioral changes (e.g., parasite-driven fish behavior or “the zombie ant fungus,” Ophiocordyceps unilateralis) (13, 14). While a number of studies have investigated this possibility, with reports in both humans and rodents linking T. gondii infection to a wide range of behavioral modifications and, more recently, neurodegenerative disease, conflicting data also exist. These contradictory findings make it unclear as to what extent T. gondii affects host behavior or secondary disease. Below, we will review a sample of key studies that exemplify the data for and against latent T. gondii to modify host behavior and outcomes.

## T. GONDII EFFECTS ON RODENT MODELS

A variety of behavioral abnormalities has been reported in T. gondii-infected rodents. Memory deficits were the first behavioral abnormality linked to T. gondii, beginning with an observational study in wild rats, which was later substantiated by experimental studies in both mice and rats (15–17). Motor abnormalities have also been reported, which include both increased activity (18–25) and decreased activity (16, 23, 26–29). Finally, infected rodents have also shown diminished fear responses as assessed by several assays, including (i) loss of aversion to cat urine (22, 30–34), (ii) decreased fear of open spaces (18, 35), and (iii) increased likelihood of eating novel food (20). Tests used for memory, fear, and motor abnormalities are listed in Table 1. Decreased fear, especially to cat urine, has been postulated to increase the chance of predation of rodents by the definitive host, thus offering a potentially plausible reason for T. gondii infection to alter this behavior.

**TABLE 1:** List of tested behaviors and methods^*a*^

Behavior	Test method
Motor activity	Deep maze
	Social interaction test
	Open field
	Elevated plus maze
Memory	Deep-maze retention
	Spontaneous alteration in Y maze
	Barnes maze short-term memory
	STFP^*b*^, olfactory memory
	Morris water maze
	Novel object recognition
	Object placement
Fear	Cat urine, cat fur
	Bobcat urine versus mink or rabbit urine
	In square pen or rectangular arena

a For a comprehensive overview, see Worth et al. (17).

b STFP, Social transmission of food preference.

While these studies suggesting a strong effect of T. gondii infection on behavior have garnered attention in both the scientific community and in the general press, it is important to note that negative studies also exist. A subset of studies reported no changes in memory (22, 29, 32, 36), no motor abnormalities (20–22, 26–28, 32, 36), and no fear differences associated with T. gondii infection (22, 23, 31, 32). Even within those studies reporting positive results, the results are more heterogenous than often recognized, with most studies reporting a mix of both positive and negative results. For instance, one study found that infected rats that showed decreased fear in one context (feline urine) had normal fear responses in another scenario (feline fur odor) (32). In addition, a more recent study reported a lack of fear response to predator and nonpredator urine, suggesting that this behavioral modification might be more generalized and not based on predation risks (37).

A range of factors—including how a given behavior was assayed or when behavior testing was performed—may account for these contradictory findings. For example, a number of different tasks have been used to assess memory performance with variable results. These tasks include performance on the deep maze (16), Barnes maze (36), Y maze (22), and the Morris water maze (32). In addition, the genotype of the infecting T. gondii strain may also affect behavioral outcomes. For example, a study that utilized two different type II/haplotype 2 strains (Me49 and Prugniaud [Pru]) that differ genetically only by <0.5% (38) identified memory impairments in rodents infected with Me49 but not with Pru (22). Similarly, a variety of rodent strains have been used, which might also impact behavioral phenotypes (see neurotransmitter studies below). Studies that reported a lack of aversion to cat urine were conducted between 4 and 16 weeks postinfection (23, 32, 39), while negative studies that reported normal aversion ranged from 4 weeks to as long as 28 weeks postinfection (22, 23, 31, 32). Finally, the method through which rodents were infected (e.g., intraperitoneally, congenitally, or orally) also fluctuates between studies, which in turn can affect host immune response and thus, potentially, outcomes of infection (40). Overall, such study heterogeneity likely contributes to the conflicting behavioral outcomes.

In addition to T. gondii’s effect on rodent behavior, a small number of studies examined T. gondii’s effect on secondary CNS insults. Mice infected with type II (Me49) T. gondii had decreased stroke sizes compared to those in uninfected mice. This protection against stroke was thought to be secondary to a diminished stroke-related immune response and increased oxidative capacity in chronically infected mice (41). More recently, three studies in three different Alzheimer’s disease mouse models that overexpress mutant human amyloid precursor protein determined that infection with a type II strain (Me49 in two studies, Pru in one study) was protective against amyloid beta (Aβ) deposition (42–44). One of the studies also documented prevention of memory deficits (42) and one study showed that protection appeared to be strain specific as infection with a type III strain did not decrease Aβ deposition (44). The postulated mechanisms for this protection include the induction of anti-inflammatory cytokines (interleukin 10 [IL-10] and transforming growth factor β [TGF-β]) (42) and an increase in infiltrating phagocytic immune cells (43) in infected mice. In summary, in a very limited number of studies in mice, type II infection appears to be protective against secondary CNS insults, suggesting that we may be able to use T. gondii infection to find new pathways for treating other neurologic diseases.

## MECHANISMS BY WHICH T. GONDII MIGHT AFFECT RODENT BEHAVIOR

Though the extent to which T. gondii infection universally influences rodent behavior is unclear, several mechanisms have been proposed to account for the observed behavioral changes. Possible mechanisms include variations in secreted neurotransmitters, the neuroanatomic location of T. gondii cysts, or modulation of the CNS by the neuroinflammatory response (34, 45).

The modulation of neurotransmitters represents an obvious mechanism for altering behavior, as these molecules are responsible for the cell-to-cell communication that drives CNS circuits and behavior. An early study focused on examining changes in dopamine (DA), norepinephrine (NE), and serotonin (5-HT) in acute and chronically infected mice because these neurotransmitters are involved in learning, memory, fear modulation, and mood (46–48). The study found that NE decreased in an acute infection and DA increased in chronically infected mice (49). These findings were replicated by a more recent study that reported similar changes in neurotransmitter levels in whole brain homogenates as well as positive behavioral changes in mice, though it is unclear if the neurotransmitter levels were measured in the same mice that underwent behavioral testing (50). One proposed mechanism for these changes is a downregulation of dopamine β-hydroxylase (DBH), an enzyme that converts DA to NE. Consistent with this hypothesis, in a recent study, NE protein and DBH mRNA were decreased in male rats, but not female rats, though neither group showed behavior changes compared to uninfected rats. The study then moved to mice, where infected male mice displayed behavioral abnormalities and decreased DBH mRNA, while infected female mice had no changes in DBH mRNA; it is unclear if behavior changes were observed in the infected female mice. In addition, the relevant neurotransmitter levels (i.e., DA and NE) were not assayed in the male or female mice (51). Another study, investigating DA, DA metabolites, NE, and 5-HT found increased levels of DA metabolites (but not DA itself) and NE in the cortices of infected mice as well as decreased 5-HT in the amygdala (52). In this study, DA metabolite levels were correlated with freezing behaviors. It is important to note, however, that a separate study that used the same strain of T. gondii but a different mouse strain found no behavior abnormality and no difference in DA levels (53). Given that immune cells make and release DA (54), the opposing outcomes between these studies may arise from the positive study using a mouse strain that develops high levels of neuroinflammation (C57BL/6 mice) while the negative study used a mouse strain that generates very little neuroinflammation (CD-1 mice). Finally, a single study has shown that glutamate levels are increased in infected mice secondary to a decrease in expression of astrocytic GLT-1, the transporter that removes glutamate released by neurons. In this study, ceftriaxone treatment restored levels of astrocytic GLT-1 and normalized brain glutamate levels but had no effects on T. gondii cyst burden, the neuroinflammatory response, or the identified behavioral abnormalities (35). These findings suggest that glutamate, the major excitatory neurotransmitter in the cortex, does not drive the T. gondii-associated behavioral abnormalities identified in this study.

The second commonly postulated mechanism for T. gondii-associated rodent behavioral changes is that the prevalence and neuroanatomical location of cysts dictate both the type and magnitude of behavioral changes that accompany infection. Again, this hypothesis makes intuitive sense, as different brain regions mediate specific functions (e.g., hippocampus and memory; occipital cortex and vision). Despite its appeal, there is very little evidence to support this hypothesis: cyst location remains highly variable from mouse to mouse, with no preponderance of cysts in brain regions implicated in behavioral changes (e.g., motor cortex or limbic system) (55). In addition, a lack of fear aversion to feline urine was reported in mice infected with a T. gondii strain that does not produce cysts (39), making the cyst hypothesis very unlikely as a cause of the altered behaviors in those mice. Finally, a very recent study showed that the administration of the drug guanabenz normalized infection-associated hyperactivity in infected C57BL/6 mice without affecting cyst number (25).

A related hypothesis is that cysts cause a local increase in DA, which in turn drives behavioral changes. This idea arose because T. gondii contains two amino hydroxylases, AAH1 and AAH2, which could convert tyrosine to L-dopa, the rate limiting step in DA synthesis (56, 57). AAH1 is an essential gene that is expressed in both tachyzoites and bradyzoites, while AAH2 is expressed only in bradyzoites and amenable to gene deletion. Three studies using two independently generated AAH2 knockout (KO) strains have laid this theory to rest, as all three studies found that mice infected with either parental or AAH2 KO T. gondii parasites showed the same behavioral changes or neurotransmitter changes regardless of the infecting T. gondii strain (24, 53, 58).

A final common hypothesis for T. gondii-associated behavioral change is that the neuroinflammatory response drives these changes. This possibility is consistent with the previously mentioned study that reported behavioral changes in association with a T. gondii strain that does not form cysts but does provokes a neuroinflammatory response (39). This hypothesis is also consistent with the guanabenz study. In this study, which used both BALB/c and C57BL/6 mice, drug treatment rescued T. gondii-associated behavioral deficits and decreased the neuroinflammatory response in both mouse strains but only decreased the cyst burden in BALB/c mice while increasing the cyst burden in C57BL/6 mice (25).

## T. GONDII AND HUMANS

The first study to link human behavior to T. gondii seropositivity found that men positive for IgG antibodies fell within distinct personality categories compared to those for uninfected controls (59). Since the original study, well over 200 papers have been published on T. gondii infection and its influence on human behavior and secondary disease. Before we review the data, we will first outline several caveats that are important to consider when evaluating these studies. We will then briefly review the data correlating T. gondii infection to personality and risky behavior, mental health disorders (e.g., schizophrenia), neurodegeneration (e.g., Parkinson’s disease), and cognition.

While rodent studies allow one to experimentally manipulate which animals are infected, human studies rely on using serologic testing (e.g., antibodies to T. gondii) to identify latently infected persons and then compare a given behavior between the seropositive and seronegative groups. Consequently, such approaches only allow for investigations of the correlation between T. gondii infection (or really, seropositivity) and behavior but cannot directly assess the causal role that infection may play in a given behavior. This difference between correlation and causality is important, because it raises a key question: does a given behavior or disease increase the likelihood of T. gondii infection rather than the other way around? At present, the published studies cannot address this critical question. In addition, serology (IgG positivity usually) does not distinguish prior exposure from ongoing chronic infection, a point highlighted by an autopsy study which found no histopathologic evidence of T. gondii in seropositive patients with behavioral abnormalities (60).

Some of the human studies are also hampered by other limitations inherent to their design. For instance, many utilize survey responses and voluntary correspondence, which decrease the sample size and may lead to sampling bias. Additionally, in places with low T. gondii seropositivity, large statistical effects can be driven by very few subjects. Reporting bias may also be a problem, as small studies with positive findings are more numerous, while negative findings are largely only published from large census data. Furthermore, many of these studies do not perform multivariate analyses or ensure that the baseline groups are matched. Finally, akin to the limitations described for the rodent studies, there is marked heterogeneity in the behavioral assays employed in investigating T. gondii-associated behavioral or cognitive change in humans.

### Personality and risky behavior.

Differences in personality types in T. gondii-seropositive individuals were the first reported change in otherwise healthy individuals, leading to a multitude of follow-up studies (59, 61–67). Since then, various other behaviors have been associated with T. gondii infection, including increased aggression (65, 68), increased risk for motor vehicle accidents (65, 69–72), decreased reaction time (73, 74), and excessive ethanol consumption (75). While risky behavior is a popular subject (76, 77), publication bias is likely a contributing factor, and most studies lack multifactorial analyses. Notably, in a population-representative birth cohort that examined poor impulse control, personality, and cognition, only suicide maintained a statistically significant correlation with T. gondii seropositivity (78).

### Mental health.

While T. gondii seropositivity has been studied in association with a number of mental health disorders, the association between T. gondii seropositivity or serointensity (the amount of IgG antibody) (79) and schizophrenia has been the most studied and publicized. While most studies focus on seroprevalence, it is often the case that if no correlation is found between seroprevalence and schizophrenia, serointensity is then considered and reported (80). A recent meta-analysis by Sutterland et al. (77) examined the association between T. gondii and mental health disorders. In this analysis, studies were only included if the original publication was in English, French, or Dutch; healthy subjects were used as controls, patients were diagnosed according to the diagnostic and statistical manual of mental disorders, third edition (DSM-III) or a higher standard, and seropositivity was determined by well-established diagnostic tests. Of the 50 studies that met these criteria, 42 were for schizophrenia. Overall, they found a significant association between T. gondii seroprevalence and schizophrenia (odds ratio [OR], 1.81) but noted that serointensity was heterogenous and results were region specific (77). Considerations for age (seroprevalence generally increases with age) and publication bias lowered the OR to 1.43, meaning that those who were seropositive for T. gondii IgG were 1.43 times more likely to also be diagnosed with schizophrenia than seronegative controls. For context, a maternal family history of schizophrenia has an OR of 9.31, paternal age >54 years has an OR as high as 5.92, and cannabis use has a reported OR of 2.93 (81). As noted above, these studies cannot determine if T. gondii infection caused or increased the likelihood of developing schizophrenia or if high-risk behaviors associated with schizophrenia increase the likelihood of becoming infected with T. gondii.

In addition to schizophrenia, bipolar disorder has also been commonly studied in association with T. gondii seropositivity, though there are far fewer published studies. In a recent meta-analysis of 118 studies on T. gondii and bipolar disorder, only 8 met their criteria: original articles, published from January 1980 to June 2015, in English, Portuguese, or Spanish, and measured T. gondii serology. This study concluded that patients with bipolar disorder were more likely to be infected by T. gondii but with a low OR of 1.26, which was likely driven by the substantial overlap in T. gondii seropositivity prevalence in the included studies: 15.6% to 95.3% in bipolar patients and 16.3% to 87.3% in controls (82).

### Neurodegeneration and cognition.

The association between T. gondii and dementia has been investigated in Parkinson’s disease (PD), Alzheimer’s disease (AD), and overall cognition. Several publications reported a positive correlation between PD and T. gondii seropositivity (83, 84), although they were quickly followed by negative studies (85, 86). In a recent meta-analysis on PD and T. gondii seropositivity, Zhou et al. included seven studies with 1,086 subjects total and found no correlation between PD and T. gondii seropositivity (87). This meta-analysis highlighted that the initial positive studies for PD had small sample sizes and likely some degree of sampling bias related to the geographical region in which the studies were conducted.

The investigation between Alzheimer’s disease (AD) and T. gondii has been driven, in part, by the idea that CNS pathogens may be drivers or potentiators of AD pathology. This concept arose for several reasons: (i) the neuropathological findings of AD (neurofibrillary tangles and amyloid beta plaques) can be seen in CNS infections; (ii) amyloid-beta has been shown to have antimicrobial properties; and (iii) DNA from a variety of pathogens has been found in the brains of AD patients (88, 89). Collectively, these findings suggested that amyloid beta accumulation might occur in response to pathogens and then cause deleterious cognitive effects.

A recent meta-analysis reviewed 8 AD studies for a total of 3,239 subjects across seven countries (90). They excluded all study types except for cross-sectional (*n* = 2) and case-control (*n* = 6) studies. Only two of the eight studies reported a significance in T. gondii seroprevalence and AD. One of the positive studies had a very high OR (49.9), but this high OR was driven by the fact that the study included only 2 patients with AD, both of whom were T. gondii seropositive, while the control population (*n* = 180) had a seroprevalence rate of 9%. The remaining studies had ORs ranging from 0.94 to 5.75 with *P* values that ranged from 0.18 to 0.99. However, the combined analysis of all 8 studies resulted in a common OR of 1.53, though the authors note that this OR may be influenced by a publication bias for positive results.

Finally, we will conclude with a review of the National Health and Nutrition Examination Survey (NHANES) III from the Centers for Disease Control because of the size and long-term follow-up of the studied cohort. This cohort has been used in multiple studies correlating T. gondii serology to cognition. A variety of cognitive tests were recorded from 4,178 participants, ages 20 to 59 years, from 1966 to 1994, in which 19.1% were seropositive for T. gondii. The breadth of this cohort allowed for many correlations between host behaviors and other factors with T. gondii seropositivity. These studies nicely illustrate the impact of multivariate analyses. While at first glance, seropositivity was correlated with decreased cognitive performance in the total population, when a multivariate analysis was performed, the positive correlation became smaller and was more associated with socioeconomic and immigration status (91, 92). Multivariate analysis allows us to have additional insights into what other factors may be driving the correlation with T. gondii seropositivity and the NHANES studies demonstrate the utility of rigorously designed and analyzed studies.

In summary, the validity of seropositivity, population incidence rates, study design limitations, and publication bias all must be considered when evaluating the link between T. gondii infection and human behavior and disease.

## DISCUSSION

Toxoplasma gondii is a unique parasite in that it causes a long-term latent infection in the CNS of humans and rodents. As such, much work has been devoted to determining if infection with T. gondii can lead to changes in rodent and human behavior and cognition and, more recently, neurodegeneration. The rodent work shows heterogeneity, with studies suggesting a positive linkage and studies showing no linkage, a finding that is also true for studies investigating the mechanisms postulated to underlie these behavioral changes—alterations in neurotransmitter release, cyst location, and neuroinflammation. As discussed in detail above, this heterogeneity may arise from the many important differences between these studies, which include differences in the behavioral assays, the timing of the behavioral assays, and the T. gondii and rodent strains used. Collectively, these data suggest that T. gondii infection does not universally cause changes in rodent behaviors and that the underlying mechanism(s) for the observed behavioral changes remains unclear.

Akin to the rodent work, the human studies also show a great deal of heterogeneity. Unlike the rodent work, in which causality can be established, human studies rely on correlating T. gondii seropositivity and behavioral or cognitive outcomes. In addition to the lack of causality, many of the human studies are confounded by issues such as small sample sizes and a lack of multivariate analyses. Overall, these conflicting data and study limitations make drawing definitive conclusions difficult, though the relatively low ORs identified in meta-analyses suggest that T. gondii’s effect on human behavior or disease may be quite mild. Consistent with T. gondii playing a limited role in modifying human behavior and disease, prevalence rates of potentially T. gondii-associated diseases (e.g., schizophrenia and Alzheimer’s disease) do not vary across countries with high and low T. gondii seropositivity (93–95). In fact, among developed countries, the United States has one of the highest prevalence and incidence rates for AD at age 65, with 4.9% and 10.5%, respectively, but one of the lower rates of T. gondii seropositivity (∼10%) (94, 95). In summary, the current data suggest that if T. gondii influences human behavior or disease, the effect is likely subtle and/or maybe highly dependent on the genetic background of the individual or the context of the infection (e.g., T. gondii strain type, route of infection, and how long the individual has been infected).
